# B-Cell Epitope Mapping of the *Plasmodium falciparum* Malaria Vaccine Candidate GMZ2.6c in a Naturally Exposed Population of the Brazilian Amazon

**DOI:** 10.3390/vaccines11020446

**Published:** 2023-02-15

**Authors:** Barbara de Oliveira Baptista, Ana Beatriz Lopes de Souza, Luana Santos de Oliveira, Hugo Amorim dos Santos de Souza, Jenifer Peixoto de Barros, Lucas Tavares de Queiroz, Rodrigo Medeiros de Souza, Linda Eva Amoah, Susheel Kumar Singh, Michael Theisen, Rodrigo Nunes Rodrigues-da-Silva, Evelyn Kety Pratt Riccio, Paulo Renato Rivas Totino, Josué da Costa Lima-Junior, Cláudio Tadeu Daniel-Ribeiro, Lilian Rose Pratt-Riccio

**Affiliations:** 1 Laboratório de Pesquisa em Malária, Instituto Oswaldo Cruz (IOC), Fundação Oswaldo Cruz (Fiocruz), Rio de Janeiro 21040-900, RJ, Brazil; 2 Centro de Pesquisa, Diagnóstico e Treinamento em Malária (CPD-Mal), Fiocruz e Secretaria de Vigilância em Saúde, Ministério da Saúde, Rio de Janeiro 21040-900, RJ, Brazil; 3 Centro de Pesquisa em Doenças Infecciosas, Universidade Federal do Acre–Campus Floresta (UFAC), Cruzeiro do Sul 69895-000, AC, Brazil; 4 Immunology Department, Noguchi Memorial Institute for Medical Research, University of Ghana, Accra P.O. Box LG 25, Ghana; 5 Centre for Medical Parasitology, Department of International Health, Immunology and Microbiology, University of Copenhagen, DK-2200 Copenhagen, Denmark; 6 Department of Infectious Disease, Copenhagen University Hospital, DK-2200 Copenhagen, Denmark; 7 Laboratório de Tecnologia Imunológica, Instituto de Tecnologia em Imunobiológicos (Bio-Manguinhos), Fiocruz, Rio de Janeiro 21040-900, RJ, Brazil; 8 Laboratório de Imunoparasitologia, IOC, Fiocruz, Rio de Janeiro 21040-900, RJ, Brazil

**Keywords:** GMZ2.6c, epitope mapping, antibodies, malaria, *Plasmodium falciparum*, vaccine

## Abstract

The GMZ2.6c malaria vaccine candidate is a multi-stage *P. falciparum* chimeric protein that contains a fragment of the sexual-stage *Pf*s48/45-6C protein genetically fused to GMZ2, an asexual-stage vaccine construction consisting of the N-terminal region of the glutamate-rich protein (GLURP) and the C-terminal region of the merozoite surface protein-3 (MSP-3). Previous studies showed that GMZ2.6c is widely recognized by antibodies from Brazilian exposed individuals and that its components are immunogenic in natural infection by *P. falciparum*. In addition, anti-GMZ2.6c antibodies increase with exposure to infection and may contribute to parasite immunity. Therefore, identifying epitopes of proteins recognized by antibodies may be an important tool for understanding protective immunity. Herein, we identify and validate the B-cell epitopes of GMZ2.6c as immunogenic and immunodominant in individuals exposed to malaria living in endemic areas of the Brazilian Amazon. Specific IgG antibodies and subclasses against MSP-3, GLURP, and *Pfs48*/45 epitopes were detected by ELISA using synthetic peptides corresponding to B-cell epitopes previously described for MSP-3 and GLURP or identified by BepiPred for *Pf*s48/45. The results showed that the immunodominant epitopes were P11 from GLURP and MSP-3c and DG210 from MSP-3. The IgG1 and IgG3 subclasses were preferentially induced against these epitopes, supporting previous studies that these proteins are targets for cytophilic antibodies, important for the acquisition of protective immunity. Most individuals presented detectable IgG antibodies against *Pf*s48/45a and/or *Pf*s48/45b, validating the prediction of linear B-cell epitopes. The higher frequency and antibody levels against different epitopes from GLURP, MSP-3, and *Pf*s48/45 provide additional information that may suggest the relevance of GMZ2.6c as a multi-stage malaria vaccine candidate.

## 1. Introduction

Malaria, an infectious disease caused by *Plasmodium* parasites, remains a major public health problem worldwide. In 2021, 247 million cases and 619.000 deaths due to malaria were estimated globally, most of them caused by *P. falciparum* [1]. Despite the remarkable progress in control over the last few years, the emergence of resistant parasites to artemisinin-based combination therapies (ACTs), the first line of treatment for uncomplicated *P. falciparum* malaria in all endemic countries [2,3,4,5,6], and resistance of *Anopheles* to available insecticides [7] highlights the need for an effective vaccine to be implemented, complementing the existing tools. Recently, the World Health Organization (WHO) recommended the widespread use of the RTS,S/AS01 vaccine among children in regions with moderate to high *P. falciparum* transmission [1]. Despite its partial and low efficacy, this vaccine might prevent about 30% of child deaths [8]. However, vaccine candidates with a higher protective efficacy should be developed. Considering the complex life cycle, an ideal malaria vaccine should be directed against antigens expressed in different parasite development stages.

The GMZ2.6c malaria vaccine candidate is a multi-stage *P. falciparum* chimeric protein that contains a fragment of the sexual-stage *Pf*s48/45-6C protein genetically fused to GMZ2, an asexual-stage vaccine construct consisting of conserved domains of glutamate-rich protein (GLURP) and merozoite surface protein-3 (MSP-3) [9]. GLURP is expressed in all parasite life cycle stages in the vertebrate host and may contribute to merozoite invasion and formation of the parasitophorous vacuole [10,11], MSP-3 is expressed in the erythrocytic stage and is involved in the invasion of red blood cells, as well as in parasite protection against heme released from unprocessed hemoglobin released after schizont egress [12,13], and *Pf*s48/45 is expressed on the surface of gametocytes and gametes, playing a central role in fertilization [14]. Both GLURP and MSP-3 are targets of cytophilic IgG subclasses (IgG1 and IgG3) able to control *P. falciparum* growth in vitro by opsonic phagocytosis [15] and antibody-dependent cellular inhibition (ADCI) [16,17,18] correlated to protection against high parasitemia and clinical disease in individuals living in endemic areas, while naturally acquired antibodies against *Pf*s48/45 can recognize extra-erythrocytic gametes, reducing transmission by preventing fertilization and sporogonic development in the mosquito midgut [19].

Previous studies showed that GMZ2.6c protein was widely recognized by naturally acquired antibodies from individuals of Brazilian endemic areas and that its components (MSP-3, GLURP, and *Pf*s48/45) are immunogenic in natural infection by *P. falciparum.* Moreover, anti-GMZ2.6c antibodies seem to increase with exposure to malaria infection and may contribute to parasite immunity [20]. Considering that antibodies recognize several antigenic determinants of the protein, identifying the contribution of different immunodominant B-cell epitopes of antimalarial vaccine candidates that induce specific immune responses may be an important tool for understanding protective immunity. Therefore, the present work aimed to identify and validate immunodominant B-cell epitopes of the *P. falciparum* chimeric protein GMZ2.6c in individuals exposed to malaria living in the Brazilian Amazon.

## 2. Materials and Methods

### 2.1. Study Area and Volunteers

A cross-sectional cohort study was carried out from June to August 2016 and 2018 in three malaria-endemic areas of the Brazilian Amazon: Cruzeiro do Sul (07°37′50″ S/72°40′13″ W) and Mâncio Lima (07°36′49″ S/72°53′47″ W), both high-risk areas situated in Juruá Valley, Acre State, the main *P. falciparum* malaria hotspot in Brazil, and Guajará (02°58′18″ S/57°40′38″ W), a medium-risk area situated in Amazonas State. Cruzeiro do Sul, Mâncio Lima, and Guajará registered 5.447, 1.432, and 674 cases in 2016, accounting for 52.6% of all *P. falciparum* infections in Brazil. Due to the increase in *P. falciparum* cases in other municipalities, this number decreased to 24.1% in 2018, with 2.915, 1.719, and 452 *P. falciparum* infections in Cruzeiro do Sul, Mâncio Lima, and Guajará, respectively [21].

Serum samples from 303 malaria-exposed individuals identified as responders to GLURP_27−500_ (*n =* 258), MSP-3_155–249_ (*n =* 170), and/or *Pf*s48/45_291–428_ (*n =* 108) recombinants (Table S1), as previously described [20], were used to investigate specific naturally acquired antibody response to GLURP, MSP-3, and *Pf*s48/45 epitopes. In addition, serum samples from 5 individuals of the laboratory staff (Rio de Janeiro, Brazil) who had neither history of malaria nor contact with malaria transmission areas were included in our study (Rio de Janeiro controls).

### 2.2. Epidemiological Survey

Donors providing informed consent answered an epidemiological survey. To evaluate the degree of malaria exposure, subjects responded to questions related to personal data such as age, time of residence in the endemic area, number of previous malaria episodes, time elapsed from the last infection, use of malaria prophylaxis, and presence of symptoms.

### 2.3. Blood Sampling and Malaria Diagnosis

Venous peripheral blood (20 mL) was collected into Heparin or EDTA tubes for antibodies analysis or molecular diagnosis, respectively. The plasma was stored at −20 °C, and the pellets, containing peripheral blood cells collected into EDTA tubes, were mixed with equal volumes of a cryopreservation solution (0.9% NaCl/4.2% sorbitol/20% glycerol) and were stored at −70 °C until use.

Thin and thick blood smears were examined for identification of malaria parasites by a technician experienced in malaria diagnosis from the Laboratory of Malaria Research (Fiocruz), which is the headquarters of the CEMART (Centre for Malaria Research and Training), a reference center for malaria diagnosis in the Extra-Amazonian Region for the Brazilian Ministry of Health. Thick blood smears from all the subjects were stained with Giemsa, and a total of 200 microscopic fields were examined under a 1000-fold magnification. Thin blood smears of the positive samples were examined for species identification. The parasite density was determined by counting the parasites in a predetermined number of white blood cells in the thick blood films, and the number of blood parasites per milliliter was calculated [22]. To increase the sensitivity of the parasite detection, molecular analysis using specific primers for genus (*Plasmodium* sp.) and species (*P. falciparum* and *P. vivax*) was performed in all samples, as previously described [23]. Positive donors for *P. vivax* and/or *P. falciparum* at the time of blood collection were subsequently treated by the chemotherapeutic regimen recommended by the Brazilian Ministry of Health [24].

### 2.4. B-Cell Epitope Prediction of Pfs48/45

The prediction of linear B-cell epitopes of *Pf*s48/45_291–428_ (UniProt: Q8I6T1) was carried out using the program BepiPred 1.0 [25]. This method is based on the combination of hidden Markov and propensity scale models, which consider hydrophilicity and secondary structure prediction. The server outputs a prediction score for each amino acid for each input FASTA sequence. To identify potential linear B-cell epitopes was used the cut-off value of 0.35, ensuring a sensibility of 49% and specificity of 75% to this approach. Linear B-cell epitopes are predicted to be located at the residues with the highest scores. Sequences with a BepiPred score above 0.35 were considered potential linear B-cell epitopes in regions that could be accessed by naturally acquired antibodies.

### 2.5. Synthetic Peptides

**Figure 1:**
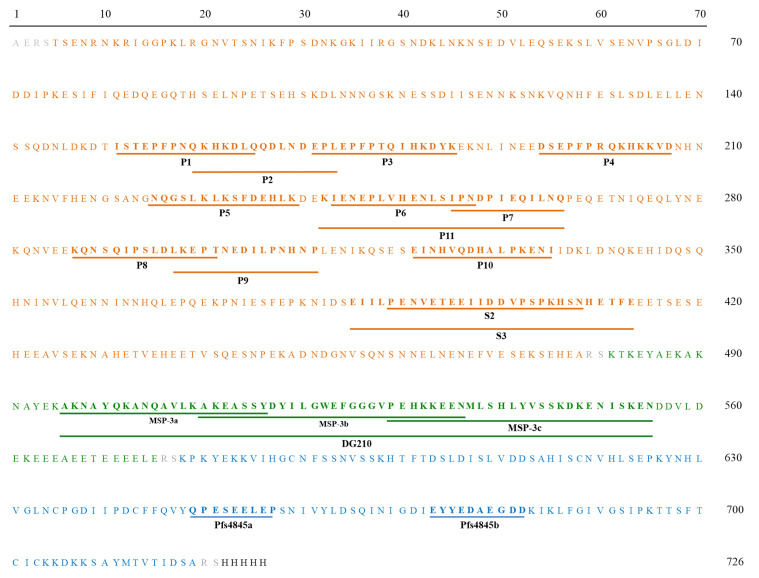
Amino acid sequence and linear epitopes of GMZ2.6c hybrid protein. Amino acid sequence of *Pf*GLURP (orange), *Pf*MSP-3 (green), and *Pf*s48/45 (blue). Bold letters represent linear epitopes. Orange, green, and blue lines represent *Pf*GLURP, *Pf*MSP-3, and *Pf*s48/45 epitopes, respectively.

B-cell epitope mapping was performed using four synthetic peptides of MSP-3_155–249_ (MSP-3a, MSP-3b, MSP-3c, and DG210) and 13 synthetic peptides of GLURP_27–500_ (P1, P2, P3, P4, P5, P6, P7, P8, P9, P10, P11, S2, and S3), previously described [26,27]. Moreover, two synthetic peptides of *Pf*s48/45_291–428_ (*Pf*s48/45a and *Pf*s48/45b) identified by prediction of linear B-cell epitopes were used (Figure 1). The selected sequences were synthesized by fluorenylmethoxycarbonyl (F-moc) solid-phase chemistry (GenOne Biotechnologies, Brazil). Analytical chromatography of the peptide demonstrated a purity of >95%.

### 2.6. Enzyme-Linked Immunosorbent Assay

Microtiter 96-well plates (Maxisorp, NUNC, Roskilde, Denmark) were coated with 5 µg/mL of each synthetic peptide in carbonate-bicarbonate buffer, pH 9.6, at 100 µL/well overnight at 4 °C. The plates were washed with phosphate-buffered saline 0.05% Tween20 (PBST), and the uncoated sites were blocked with 5% powdered milk containing PBST for 1 h at 37 °C. Serum samples diluted 1:100 in dilution buffer (1% powdered milk containing PBST) were added in duplicate wells, and plates were incubated for 1 h at 37 °C. The plates were washed, 100 µL of peroxidase-conjugated mouse anti-human IgG (Sigma, St. Louis, MO, USA) 1:1000 in dilution buffer was added, and the plates were incubated for 1 h at 37 °C. To detect specific IgG subclass, plates were incubated with peroxidase-conjugated mouse anti-human IgG1, IgG2, IgG3, and IgG4 (Clones: 4E3, 31-7-4, HP6050, and HP6025 for IgG1, IgG2, IgG3, and IgG4, respectively; SouthernBiotech, Birmingham, AL, USA) 1:1000 in dilution buffer for 1 h at 37 °C. After washing, 100 µL of a solution of 0.4 mg/mL orthophenylenediamine (OPD, Sigma) and H_2_O_2_ 30% (Sigma) in citrate-phosphate buffer (24 mM citric acid, Sigma, and 51 mM dibasic sodium phosphate, Sigma), pH 5.0, were added to each well, the plates were incubated for 5 min at room temperature in the dark, and then 50 µL/well of 2N H_2_SO_4_ (Sigma) were added. Optical density was identified at 492 nm using a SpectraMax 250 ELISA reader (Molecular Devices, Sunnyvale, CA, USA). Samples of non-endemic controls obtained from 5 individuals of the laboratory staff (Rio de Janeiro controls) were used to establish the normal range for each assay. The cut-off values were determined as the mean optical density (OD) plus 3 standard deviations of the Rio de Janeiro controls. The results were expressed as the reactivity index (RI), which was calculated by the OD mean of each tested sample divided by the cut-off value. Subjects were scored as responders if the RI against each epitope was higher than 1.0.

### 2.7. Statistical Analysis

Data were stored in the Epi-Info version 6 data bank system (Centers for Disease Control and Prevention, Atlanta, GA, USA) and analyzed using Epi-Info version 6 and GraphPad Prism (GraphPad Sftware, Inc., San Diego, CA, USA) software programs. Normality tests were performed in all variables using the one-sample Kolmogorov–Smirnov test. The chi-square analysis was applied to compare the prevalence among groups. Kruskal–Wallis, followed by uncorrected Dunn’s test (for multiples comparisons) or Mann–Whitney test (comparisons between two groups), was used to analyze differences in distributions of continuous numerical variables. The Spearman rank coefficient test was used to analyze the correlation between variables. A two-sided *p*-value of ≤0.05 was considered statistically significant.

## 3. Results

### 3.1. Population Characteristics

The studied population was composed of 303 individuals living in three malaria-endemic areas of the Brazilian Amazon (Table 1). The population age ranged from 12 to 88 years old (median: 32 years; interquartile range: 28–34) and presented a similar frequency of female (47.5%) and male (52.5%) individuals. Most participants (99%) were naturally exposed to malaria infection throughout the years (median: 31 years; interquartile range: 28–34), and 295 (97.4%) reported one or more previous malaria episodes. Among those who remembered the *Plasmodium* species, previous episodes were attributed only to *P. falciparum* (12 cases), to *P. vivax* (50 cases), or both parasites (199 cases). The number of reported past malaria episodes varied greatly among participants, ranging from 0 to 100 (median: 8; interquartile range: 6–10). The time elapsed since the last malaria infection ranged from 0 to 684 months (median: 12 months; interquartile range: 6–12). At the time of blood sampling, 145 individuals (47.8%) presented symptoms that started 1 to 30 days earlier (median: 4 days; interquartile range: 2–4), with headache, fever, and chills the most frequent ones. A total of 135 individuals (44.5%) had detectable parasites, 53 (17.5%) were infected with *P. falciparum* (median parasitemia: 8000 parasites/µL of blood; interquartile range: 4000–12,000), and 82 (27%) were infected with *P. vivax* (median parasitemia: 20,000 parasites/µL of blood; interquartile range: 7000–32,000).

### 3.2. Frequencies and IgG Levels of Pre-Identified Linear B-Cell Epitopes of GLURP_27–500_ and MSP-3_155–249_

**Figure 2:**
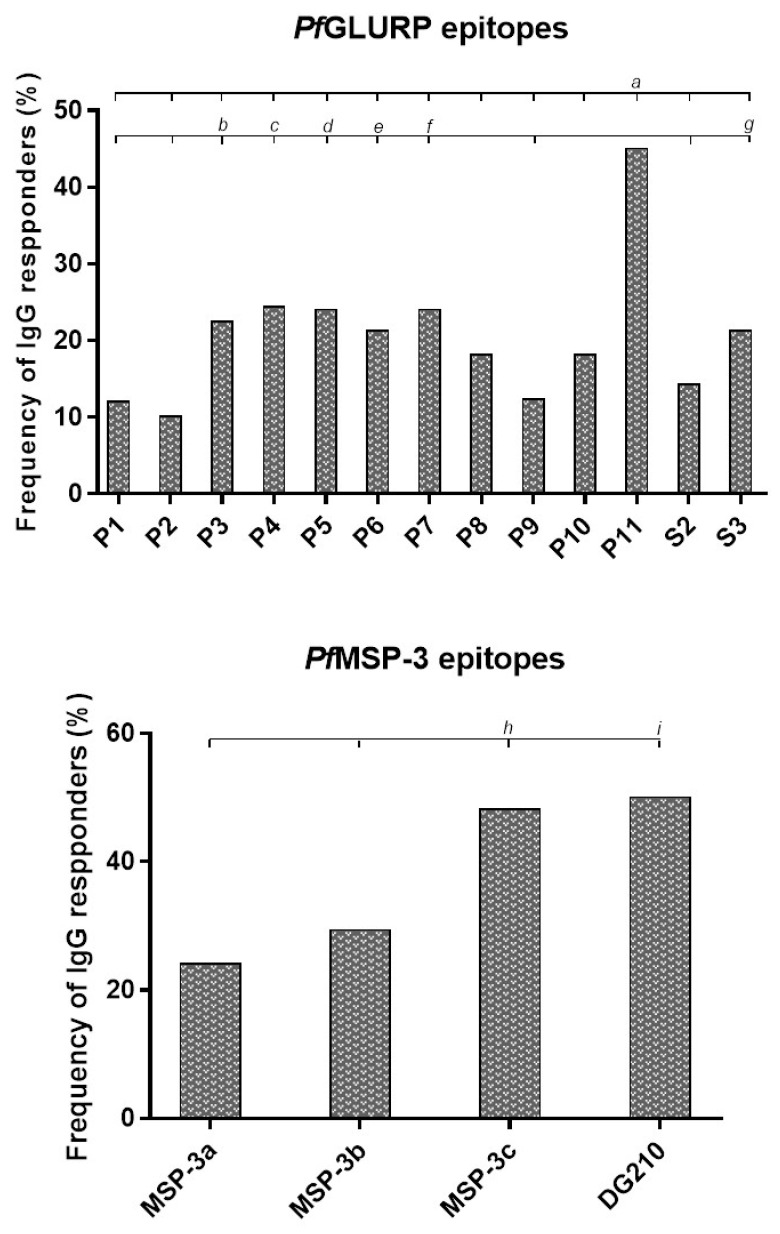
Frequency of individuals presenting IgG antibodies against epitopes derived from GLURP_27–500_, MSP-3_155–249_. Frequency of IgG responders to *Pf*GLURP and *Pf*MSP-3 epitopes. *a: p* < 0.0001 P11 versus all *Pf*GLURP epitopes; *b*: *p* < 0.05 P3 versus S2, *p* < 0.005 P3 versus P1 and P9, *p* < 0.0005 P3 versus P2; *c*: *p* < 0.005 P4 versus S2, *p* < 0.0005 P4 versus P1, P2, and P9; *d*: *p* < 0.05 P5 versus S2, *p* < 0.0005 P5 versus P1, P2, and P9; *e*: *p* < 0.05 P6 versus P9 and S2, *p* < 0.005 P6 versus P1, *p* < 0.0005 P6 versus P2; *f*: *p* < 0.05 P7 versus S2, *p* < 0.0005 P7 versus P1, P2, and P9; *g*: *p* < 0.05 S3 versus P9 and S2, *p* < 0.005 S3 versus P1, *p* < 0.0005 S3 versus P2; *h*: *p* < 0.0001 MSP-3c versus MSP-3a, *p* = 0.0004 MSP-3c versus MSP-3b; *i*: *p* < 0.0001 DG210 versus MSP-3a, *p* = 0.0001 DG210 versus MSP-3b.

Firstly, amino acid sequences from GLURP_27–500_ (P1, P2, P3, P4, P5, P6, P7, P8, P9, P10, P11, S2, and S3) and MSP-3_155–249_ (MSP-3a, MSP-3b, MSP-3c, and DG210) previously identified as B-cell epitopes to validate their immunodominance in the studied population were selected. The frequencies of individuals with IgG antibodies that recognized at least one of the peptides were 70.9% and 64.7% for GLURP and MSP-3, respectively. The frequency of IgG antibody response varied depending on the peptide, showing that P11 from GLURP_27–500_ and MSP-3c and DG210 from MSP-3_155–249_ were preferentially recognized by antibodies from exposed individuals (Figure 2). Although lower, the frequencies of individuals with IgG antibodies against epitopes P3, P4, P5, P6, P7, and S3 were higher than the frequencies of individuals with IgG antibodies against epitopes P1, P2, P9, and S2 (Figure 2).

**Figure 3:**
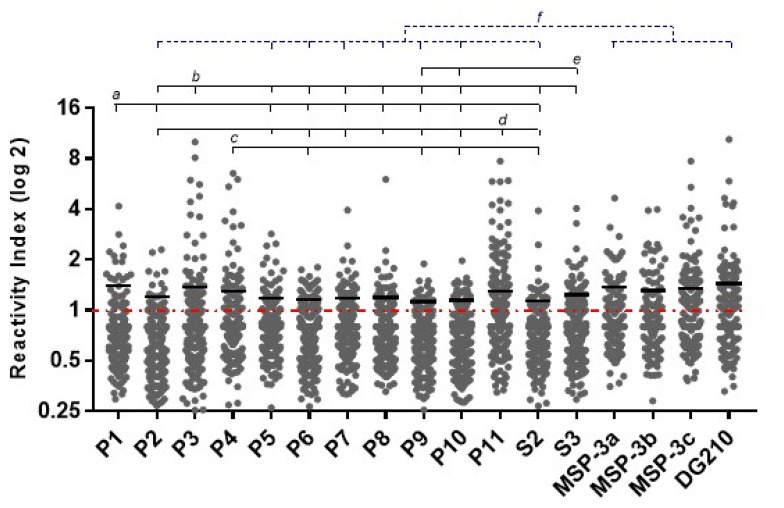
Levels of IgG antibodies (reactivity index) against GLURP_27–500_ and MSP-3_155–249_ epitopes. Reactivity indices are individual values. The dashed red line represents the positivity limit. Lines represent the median. *a*: *p* < 0.05 P1 versus P2, P5, P7, and P8; *p* < 0.005 P1 versus P6, P9, P10, and S2; *b*: *p* < 0.05 P3 versus P2 and S3; *p* < 0.005 P3 versus P5, P7, and P8; *p* < 0.0005 P3 versus P6, P9, P10, and S2; *c*: *p* < 0.05 P4 versus P6, P9, and S2; *p* < 0.005 P4 versus P10; *d*: *p* < 0.05 P11 versus P2, P5, P7, and P8; *p* < 0.005 P11 versus S2; *p* < 0.0005 P11 versus P6, P9, and P10; *e*: *p* < 0.05 S3 versus P9 and P10; *f*: *p* < 0.05 MSP-3a versus P2, P5, P7, and P8; MSP-3b versus P5 and S2; MSP-3c and DG210 versus P2; *p* < 0.005 MSP-3b versus P6, P9, and P10; MSP-3c and DG210 versus P5, P7, and P8; *p* < 0.0005 MSP-3a versus P6, P9, and P10; MSP-3c and DG210 versus P6, P9, P10, and S2.

Regarding the magnitude of response to epitopes among responders, higher levels of IgG antibodies against epitopes P1, P3, P4, P11, and S3 were observed. No difference was observed in IgG antibody levels between MSP-3_155–249_ epitopes. However, MSP-3 epitopes MSP-3a, MSP-3c, and DG210 induced higher IgG antibody levels than GLURP epitopes P2, P5, P6, P7, P8, P9, P10, and S2 (Figure 3).

No association was observed among frequency of responders or IgG antibody levels and age, sex, time of exposure, presence of symptoms, number of reported previous malaria episodes, the time elapsed since the last malaria episode, and current or last infecting plasmodial species.

### 3.3. IgG Subclasses Distribution against the Immunodominant Epitopes of GLURP_27–500_ and MSP-3_155–249_

**Figure 4:**
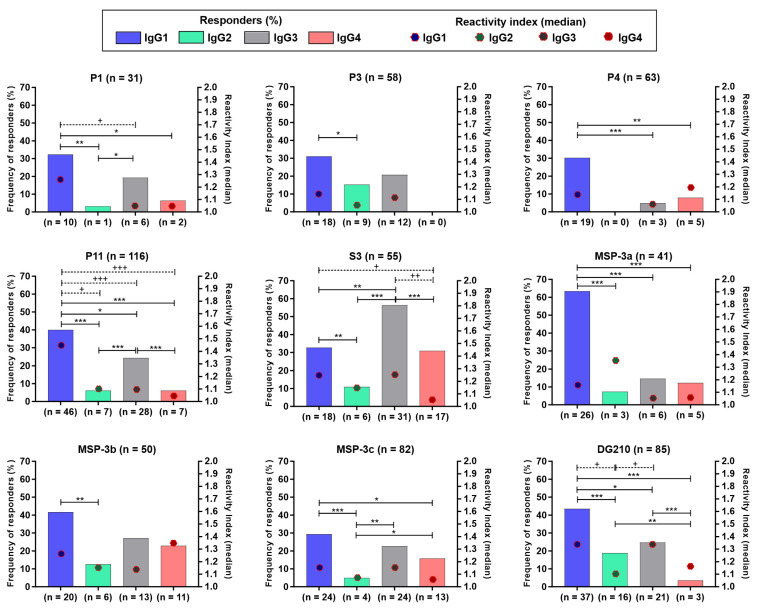
Frequency and levels of IgG subclasses against the immunodominant epitopes from GLURP_27–500_ and MSP-3_155–249_. The bars represent the frequency of responders, and the circles with broken red lines represent IgG subclasses levels (median). Significant differences among subclass frequencies were indicated by * and significant differences among subclasses levels were indicated by +; (*; +) *p* < 0.05; (**; ++) *p* < 0.005; (***; +++) *p* < 0.0005.

To evaluate the balance between cytophilic and non-cytophilic antibodies, immunodominant epitopes derived from GLURP_27–500_ (P1, P3, P4, P11, and S3) and MSP-3_155–249_ (MSP-3a, MSP-3b, MSP-3c, and DG210) were selected. The results showed that epitopes P1, P3, P11, and S3 of GLURP and MSP-3b, MSP-3c, and DG210 of MSP-3 were preferentially recognized by IgG1 and IgG3 cytophilic antibodies, while epitopes P4 of GLURP and MSP-3a of MSP-3 were preferentially recognized by antibodies of the IgG1 subclass. No difference was observed between IgG1, IgG2, IgG3, and IgG4 antibody levels in responders to P3, P4, MSP-3a, MSP-3b, and MSP-3c. However, higher IgG1 than IgG3 antibody levels against P1 and IgG1 than IgG2, IgG3, and IgG4 antibody levels against P11 were detected. In addition, IgG1 and IgG3 were higher than IgG4 antibody levels against S3 and IgG2 antibody levels against DG210 (Figure 4).

IgG1 antibody levels against P1 and P11 were positively correlated with age and time of residence in malaria-endemic area (P1: *p* = 0.002; r = 0.530; for both; P11: *p* = 0.02; r = 0.207; *p* = 0.03; r = 0.201; respectively). In addition, IgG3 antibody levels against DG210 were associated with the number of previous malaria episodes (*p* = 0.034; r = 0.240), while IgG1 antibody levels against P3 were negatively correlated with time elapsed since the last malaria episode (*p* = 0.024; r = −0.316).

### 3.4. Experimental Validation of Predicted Linear B-Cell Epitopes of Pfs48/45_291–428_

**Figure 5:**
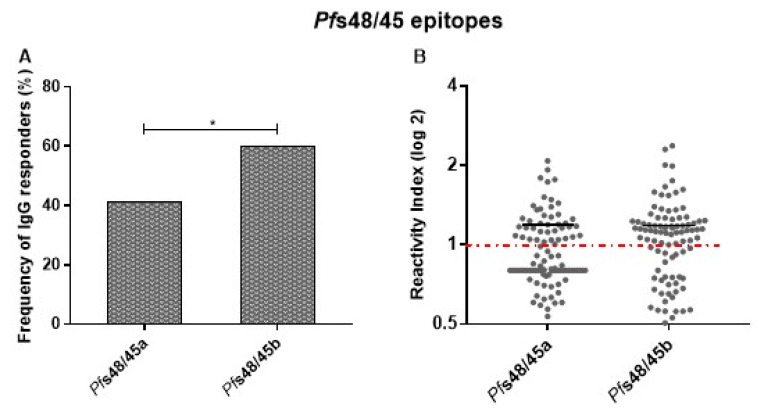
Frequency and levels of IgG antibodies against predicted *Pf*s48/45_291–428_ epitopes. (**A**) Frequencies of IgG responders to *Pf*s48/45_291–428_ epitopes. * *p* = 0.0097. (**B**) Levels of IgG antibodies to *Pf*s48/45_291–428_ epitopes. Reactivity indices are individual values. The dashed red line represents the positivity limit. Lines represent the median.

Two sequences were predicted as potential linear B-cell epitopes on *Pf*s48/45_291–428_ (*Pf*s48/45a and *Pf*s48/45b) and selected for experimental validation. Most responders to *Pf*s48/45_291–428_ recombinant protein presented detectable IgG antibodies against epitopes *Pf*s48/45a and/or *Pf*s48/45b. A higher frequency of individuals presenting IgG antibodies against *Pf*s48/45b than *Pf*s48/45a was observed. However, low IgG antibody levels were detected in epitopes, without differences between *Pf*s48/45a and *Pf*s48/45b (Figure 5).

The analyses of IgG subclasses were performed in all samples with detectable IgG antibodies against *Pf*s48/45a or *Pf*s48/45b. The frequencies of IgG1, IgG2, IgG3, and IgG4 against *Pf*s48/45a were 21%, 0%, 37% and 9%, respectively, and against *Pf*s48/45b were 43%, 11%, 26%, and 21%, respectively. The data showed a higher frequency of individuals presenting IgG3 than IgG4 antibodies against *Pf*s48/45a (*p* = 0.002) and IgG1 than IgG2, IgG3, and IgG4 antibodies against *Pf*s48/45b (*p* < 0.0001, *p* = 0.03 and *p* = 0.007 versus IgG2, IgG3, and IgG4, respectively). No difference was observed in IgG1, IgG2, IgG3, and IgG4 antibody levels against *Pf*s48/45 epitopes.

### 3.5. Frequencies and IgG Antibody Levels in Non-Infected and Infected by P. vivax and P. falciparum Individuals

Frequencies and IgG antibody levels to GLURP_27–500_, MSP-3_155–249,_ and Pfs48/45_291–428_ epitopes were compared between non-infected and infected by *P. vivax* and *P. falciparum* individuals. The results showed higher frequencies of individuals responders to P7 and P8 infected by *P. falciparum* when compared with *P. vivax*-infected individuals. Responders to S3 epitope infected by *P. vivax* were less frequent than non-infected and *P.falciparum*-infected individuals. Individuals infected by *P. falciparum* presented a higher frequency of response to MSP-3a, MSP-3b, and MSP-3c epitopes than non-infected and *P.vivax*-infected individuals, while the frequency of responders to DG210 infected by *P. falciparum* was higher than non-infected individuals (Table 2). No difference was observed in frequencies of responders to P1, P2, P3, P4, P5, P6, P9, P10, P11, S2, *Pf*s48/45a, and *Pf*s48/45b between individuals non-infected and infected by *P. vivax* or *P. falciparum*.

**Figure 6:**
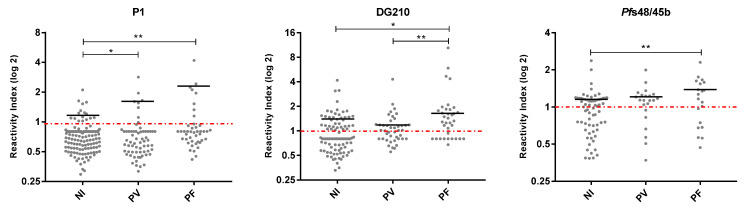
Levels of IgG antibodies (reactivity index) against P1, DG210, and Pfs48/45b epitopes in non-infected and infected by *P. vivax* and *P. falciparum* individuals. Reactivity indices are individual values. The dashed red line represents the positivity limit. Lines represent the median. NI: non-infected individuals; PV: *P. vivax*-infected individuals; PF: *P. falciparum*-infected individuals. P1: * *p* = 0.02 NI versus PV, ** *p* = 0.002 NI versus PF; DG210: * *p* = 0.01 NI versus PF, ** *p* = 0.001 PV versus PF; *Pf*s48/45b: ** *p* = 0.002 NI versus PF.

IgG antibody levels against P1 were higher in *P. vivax* and *P. falciparum*-infected than in non-infected individuals. In addition, *P. falciparum*-infected individuals presented higher IgG antibody levels than *P. vivax*-infected and non-infected individuals against DG210 than non-infected individuals against Pfs48/45b (Figure 6). No difference was observed in antibody levels against P2, P3, P4, P5, P6, P7, P8, P9, P10, P11, S2, S3, MSP-3a, MSP-3b, MSP-3c, and Pfs48/45a epitopes between non-infected and infected by *P. vivax* or *P. falciparum* individuals.

## 4. Discussion

The GMZ2.6c malaria vaccine candidate is a multi-stage *P. falciparum* chimeric protein that contains the sexual-stage *Pf*s48/45_291–428_ fragment genetically fused to GMZ2, an asexual-stage vaccine construction consisting of GLURP_27–500_ and MSP-3_155–249_ antigens [9]. The GMZ2.6c and its components were widely recognized by naturally acquired antibodies from Brazilian exposed individuals [20]. Antibodies recognize and bind their target protein antigens by surface-accessible sites, known as antigenic determinants or epitopes [28]. The identification of B-cell epitopes of proteins is an important tool for the development of epitope-based vaccines [29], diagnostic tests [30], immunotherapy [31], and understanding specific immune responses against pathogens. This work identified and validated the immunodominant B-cell epitopes of GLURP_27–500_, MSP-3_155–249,_ and *Pf*s48/45_291–428_ antigens, components of GMZ2.6c malaria vaccine candidate in individuals exposed to malaria in the Brazilian Amazon.

The studied population was composed of individuals living in three malaria-endemic areas of the Brazilian Amazon, Cruzeiro do Sul and Mâncio Lima, Acre State, and Guajará, Amazonas State. The highly variable range of age, time of residence in the endemic area, reported number of previous malaria episodes, and the time elapsed from the last infection indicated different degrees of exposure among the studied individuals, an important determinant of protection against clinical malaria [32,33]. Most of the individuals reported previous infections by both *P. falciparum* and *P. vivax*, but *P. vivax* was the most prevalent infecting plasmodial species, reflecting the current malaria scenario in Brazil, where 89% of the recorded cases are due to *P. vivax* infection [34].

GLURP and MSP-3 were identified as targets of naturally acquired antibodies capable of mediating parasite killing in cooperation with monocytes [35,36] associated with malaria protection in several epidemiological statuses [37,38,39,40,41,42]. Considering the protective role of antibodies against GLURP and MSP-3, the antigenicity of these proteins has been evaluated in epitope mapping studies. In the present work, 13 peptides derived from GLURP_27–500_ previously predicted as potential B-cell epitopes [27], four peptides derived from MSP-3_155–249_, a polypeptide recognized by antibodies from clinical immune individuals utilizing clones from a DNA library, and its three overlapping peptides [26,35] were used.

Epitope P11 was identified as the immunodominant of the GLURP with both higher frequency of responders and IgG antibody levels in the studied population. Likewise, P11 was identified as the immunodominant B-cell epitope of the GLURP in individuals living in Rondônia State, also in the Brazilian Amazon [43], and *Saimiri sciureus* monkeys immunized with a hybrid protein containing the R0 region of GLURP and the C-terminal region of MSP-3 [44]. In contrast, only 29% of clinical immune Liberian adults present antibodies against P11, while the immunodominant epitopes were P1, P3, P4, and S3 [27]. The differences in the immunodominance of GLURP B-cell epitopes may be due to polymorphisms in the R0 region of GLURP when isolates from different geographic areas were compared [45], genetic restriction of the antibody response against GLURP-R0 epitopes [43] or differences transmission intensities [46] between endemic areas.

Although with low frequency, the responding individuals displayed high levels of IgG antibodies against the P1, P3, P4, and S3 GLURP epitopes. Interestingly, P1, P3, and P4 contain a common motif (EPFPXQXHK) that appears to be targeted by cross-reactive antibodies [27]. Similarly, affinity-purified antibodies to S3 can recognize the S4 peptide derived from the C-terminal R2 region of GLURP [27]. This finding may suggest that the high antibody levels against these peptides could be the result of cross-reactivity.

The linear peptide containing the amino acid sequence of DG210 from MSP-3 was also shown to be widely recognized by high levels of naturally acquired IgG antibodies. Interestingly, similar frequency and antibody levels to peptide MSP-3c were found, suggesting that antibody response against DG210 may primarily target this epitope in the studied population. Many seroepidemiological studies demonstrated distinct profiles of antibody response to peptides derived from MSP-3CT. Peptide MSP-3b is the major B-cell epitope in hyperimmune individuals [26,35], while subjects with diverse degrees of exposure showed no differences between prevalence and antibody levels against MSP-3b, MSP-3c, and MSP-3d [47]. Antibodies induced after immunization with MSP-3CT LPS were directed against peptides MSP-3c and MSP-3d [48,49], but similar antibody levels against all peptides were found.

It is widely accepted that cytophilic IgG1 and IgG3 isotypes are the main mediators of the protective humoral response against *P. falciparum* blood-stage antigens, promoting Fc-mediated effector functions as antibody-dependent cellular inhibition (ADCI) [50,51], opsonic phagocytosis [52,53], antibody-dependent respiratory bursty (ADRB) [54] and complement activation [55,56]. In contrast, non-cytophilic IgG subclasses (IgG2 and IgG4) with the same specificity could block these effector mechanisms [57]. In our cohort, all the immunodominant epitopes of GLURP and MSP-3 are primarily targeted by IgG1 and/or IgG3 antibodies—predominantly IgG3 isotype against epitope S3 and IgG1 isotype against other epitopes. The prevalence of cytophilic antibodies against MSP-3 and GLURP epitopes was also observed in exposed individuals from Africa [47,58] and immunized European volunteers [48] associated with protection. It is known that genetic background, degrees of exposure of the population, and malaria transmission levels may influence the distribution of IgG subclasses against plasmodial antigens [59]. However, these data suggest that intrinsic characteristics of these antigens might be the major factor that determines antibody cytophilic IgG subclasses response, driving to B-cell activation by the binding of CD40 to CD40L expressed on the surface of activated T cells providing the costimulatory signal and cytokines release, influencing the IgG class switching [60,61].

Correlation analysis of the immune response and epidemiological data showed that IgG1 antibody levels against GLURP epitopes P1 and P11 were positively correlated with age and time of residence in malaria-endemic area, while IgG3 antibody levels against MSP-3 epitope DG210 were associated with the number of previous malaria episodes. In addition, IgG1 antibody levels against GLURP epitope P3 were negatively correlated with time elapsed since the last malaria episode. These may reflect the cumulative exposure of these individuals to *P. falciparum* infections and possibly maturation of the immune system over time [17] but did not provide evidence of a possible protective role of these antibodies since no relationship was verified between the prevalence or levels of specific antibodies and the presence or absence of parasites in the blood, the presence or absence of symptoms at the time of collection, and parasitemia. A detailed evaluation of the functional activity of specific antibodies against these peptides is currently being conducted in our laboratory and may generate further evidence of their possible protective role.

The *Pf*s48/45 antigen is one of the most well-characterized antibody targets of *P. falciparum* gametocytes. Several studies showed that antibodies against *Pf*s48/45 displayed a transmission reduction activity in the standard membrane-feeding assay (SMFA) [62,63], and antibodies against the conformational epitope I in the 6c domain of *Pf*s48/45 have strong transmission block potential in genetically diverse parasite strains and multiclonal infections [64,65]. Transmission-blocking antibodies are known to often target conformational-dependent epitopes [66], and no linear B-cell epitopes against *Pf*s48/45 have been described. Here, we identified two potential linear B-cell epitopes (*Pf*s48/45a: *Pf*s48/45_358–366_ and *Pf*s48/45b: *Pf*s48/45_382–391_) of *Pf*s48/45-6c domain using *in silico* BepiPred algorithm [25]. Most of the responders to *Pf*s48/45_291–428_ recombinant protein (68.5%) presented detectable IgG antibodies against *Pf*s48/45a and/or *Pf*s48/45b peptides, validating the prediction of linear B-cell epitopes. These frequencies are comparable to those found for GLURP_27–500_ (70.9%) and MSP-3_155–249_ (64.7%) peptides in this study, as well as other *P. falciparum* antigens [67,68,69]. A higher frequency of responders to *Pf*s48/45b was observed, despite no difference between the antibody levels against both epitopes. Interestingly, antibody levels against *Pf*s48/45a and *Pf*s48/45b were similar to those against *Pf*s48/45_291–428_ recombinant protein [20]. These findings indicate that although the minority of B-cell epitopes are linear (~10%) [70], they might be an interesting antibody target. However, the most potent transmission-blocking epitope in *Pf*s48/45-6c is conformational, and the production of a non-properly folded protein did not elicit functional antibodies in mice [71]. Further, the immunization with full-length *Pf*s48/45 has revealed that a larger number of antibodies with no transmission-blocking potential recognize the 6c domain of *Pf*s48/45 [72]. Additional studies are needed to better understand the humoral immune response against these epitopes and their possible transmission-blocking activity.

Studies have revealed that the high transmission-blocking efficacy of the monoclonal antibody 85RF45.1 is due to its ability to block the normal function of *Pf*s48/45 or its binding partners [72], and several IgG subclasses may contribute to the transmission-blocking activity. Curiously, the high frequency of IgG1 and IgG3 against Pfs48/45b and Pfs48/45a, respectively, and the less proportion of IgG2 and IgG4 antibodies to both epitopes suggest a possible role of the classical complement activation pathway. Although there is no evidence of the relevance of the anti-Pfs48/45 antibody–complement system interaction in transmission-blocking immunity, antibodies against the sexual-stage antigen Pfs230 showed an increase in transmission-blocking activity in the presence of human complement system proteins [73].

Specific antibodies against sexual-stage antigens are also related to age and serve as markers of exposure, increasing according to gametocyte densities [62,74,75], but no correlation with epidemiological parameters was found in this study. This finding is not surprising since, in malaria-endemic areas, infections are determined by microscopic parasite counts, and gametocytes normally occur at low densities. In addition, no other higher-sensitive method to detect gametocytes was used.

In conclusion, the data presented here showed that P11 from GLURP, MSP-3c, and DG210 from MSP-3, and the predicted epitopes Pfs48/45a and Pfs48/45b from Pfs48/45_291_ were widely recognized by antibodies from individuals living in endemic areas of Brazil. Combined immunoinformatic and experimental approach strategies identified several linear B-cell epitopes from GLURP_27–500_, MSP-3_155–249_, and Pfs48/45_291–428_ fragments that composed GMZ2.6c, providing additional information that may suggest its relevance as a multi-stage malaria vaccine candidate. Further studies are needed to assess the potential of specific antibodies against GMZ2.6c epitopes in the inhibition of *P. falciparum* growth and transmission-blocking activity.

## Figures and Tables

**Table 1:** The studied population’s personal, clinical, and epidemiological characteristics.

Personal Data		*n* = 303
Sex	Male	159/303 (52.5%)
	Female	144/303 (47.5%)
Age (years)		32 (28–34)
Time of residence in malaria-endemic area (years)		31 (28–34)
Clinical And Epidemiological Data		
Number of past malaria episodes		8 (6–10)
Time elapsed since the last malaria episode (months)		12 (6–12)
Time of symptoms (days)		4 (2–4)
Diagnosis	*P. falciparum*	53 (17.5%)
	*P. vivax*	82 (27%)
Parasitemia (parasites/µL of blood)	*P. falciparum*	8000 (4000–12,000)
	*P. vivax*	20,000 (7000–32,000)

Age, time of residence in malaria-endemic area (years), number of past malaria episodes, time elapsed since the last malaria episode (months), time of symptoms (days), and parasitemia (parasites/µL of blood) are represented by median (interquartile range). *n*: number; %: percentage.

**Table 2:** IgG antibody response in non-infected and *P. vivax-* or *P. falciparum*-infected individuals.

		NI	PV	PF
P7	Responders	37/148 (25%)	10/65 (15.4%)	15/45 (33.3%) ^a^
	Non-responders	111/148 (75%)	55/65 (84.6%)	30/45 (66.7%)
P8	Responders	30/152 (19.7%)	6/63 (9.5%)	11/43 (25.6%) ^b^
	Non-responders	122/152 (80.3%)	57/63 (90.5%)	32/43 (74.4%)
S3	Responders	41/161 (25.5%)	3/61 (4.9%) ^c^	11/36 (30.6%)
	Non-responders	120/161 (74.5%)	58/61 (95.1%)	25/36 (69.4%)
MSP-3a	Responders	15/91 (16.5%)	6/43 (14%)	20/36 (55.6%) ^d^
	Non-responders	76/91 (83.5%)	37/43 (86%)	16/36 (44.4%)
MSP-3b	Responders	20/90 (22.2%)	11/44 (25%)	19/36 (52.8%) ^e^
	Non-responders	70/90 (77.8%)	33/44 (75%)	17/36 (47.2%)
MSP-3c	Responders	42/94 (44.7%)	16/42 (38.1%)	24/34 (70.6%) ^f^
	Non-responders	52/94 (55.3%)	26/42 (61.9%)	10/34 (29.4%)
DG210	Responders	38/93 (40.9%)	23/42 (54.8%)	24/35 (68.6%) ^g^
	Non-responders	55/93 (59.1%)	19/42 (45.2%)	11/35 (31.4%)

NI: non-infected individuals; PV: *P. vivax*-infected individuals; PF: *P. falciparum*-infected individuals. ^a^ *p* = 0.02 PF versus PV; ^b^ *p* = 0.02 PF versus PV; ^c^ *p* = 0.0006 NI versus PV, *p* = 0.0005 PF versus PV; ^d^ *p* < 0.0001 NI versus PF, *p* = 0.01 PV versus PF; ^e^ *p* = 0.0008 NI versus PF, *p* = 0.01 PV versus PF; ^f^ *p* = 0.009 NI versus PF, *p* = 0.004 PV versus PF; ^g^ *p* = 0.005 NI versus PF.

## Data Availability

The datasets supporting the conclusions of this article are included within the article and its Supplementary Materials.

## Supplementary Materials

The following supporting information can be downloaded at: https://www.mdpi.com/article/10.3390/vaccines11020446/s1, Table S1: IgG reactivity index for GLURP, MSP-3, and Pfs48/45 recombinant proteins.
