# Supplementary material for: B-Cell Epitope Mapping of the Plasmodium falciparum Malaria Vaccine Candidate GMZ2.6c in a Naturally Exposed Population of the Brazilian Amazon
Source: Vaccines (Basel). 2023 Feb 15;11(2):446. doi: 10.3390/vaccines11020446 (PMC9966924; doi:10.3390/vaccines11020446)
Supplement: Supplementary file 1 [file vaccines-11-00446-s001.zip › vaccines-2180832-SI.pdf]

Table S1: IgG reactivity indeces for GLURP, MSP-3 and Pfs48/45 recombinant proteins.

	IgG reactivity index		
Sample	GLURP	MSP-3	Pfs48/45
CZS 1	1.203	1.109	0.885
CZS 4	1.100	1.100	0.513
CZS 5	1.817	1.105	1.100
CZS 6	1.269	0.835	0.947
CZS 7	1.100	1.100	1.020
CZS 8	1.329	1.134	1.119
CZS 10	5.042	3.044	1.400
CZS 11	1.813	1.200	1.100
CZS 12	3.175	1.204	0.684
CZS 13	11.018	1.866	2.582
CZS 14	8.804	1.356	1.222
CZS 15	3.529	1.743	1.100
CZS 17	1.094	1.188	1.273
CZS 18	1.451	0.825	0.877
CZS 19	1.100	0.708	0.518
CZS 20	0.614	1.003	0.677
CZS 21	0.588	0.775	1.005
CZS 22	11.555	1.419	1.550
CZS 23	3.130	1.100	0.466
CZS 24	4.517	1.100	0.546
CZS 26	15.617	2.817	1.100
CZS 27	4.119	1.630	0.528
CZS 28	3.001	0.534	1.005
CZS 29	0.954	1.149	0.936
CZS 30	0.703	1.006	0.505
CZS 31	0.950	0.869	1.101
CZS 32	8.949	1.278	0.619
CZS 33	1.174	1.227	1.006
CZS 34	1.399	1.009	0.796
CZS 35	1.488	0.537	0.606
CZS 36	1.566	0.719	0.744
CZS 37	1.488	0.775	0.817
CZS 38	1.126	0.584	0.885
CZS 39	1.100	1.100	0.772
CZS 40	1.010	0.512	1.100
CZS 41	1.088	0.635	0.823
CZS 42	0.503	1.482	0.690
CZS 43	1.122	1.162	1.008
CZS 44	2.088	7.742	0.852
CZS 45	3.078	0.890	0.658
CZS 46	2.012	0.540	1.152
CZS 48	3.669	7.040	1.006
CZS 49	1.521	0.614	1.039
CZS 50	1.078	1.914	0.940
CZS 51	13.200	1.489	1.057
CZS 52	1.100	1.100	1.100
CZS 53	0.914	1.131	0.947
CZS 54	2.846	1.440	1.368
CZS 55	0.854	0.697	1.004

CZS 56	1.844	0.829	0.535
CZS 57	1.100	0.658	0.885
CZS 58	1.070	1.400	0.951
CZS 62	0.910	1.139	0.863
CZS 63	0.810	1.070	0.874
CZS 64	0.754	1.250	0.700
CZS 66	5.280	1.286	0.885
CZS 67	1.076	0.886	0.693
CZS 69	0.797	1.200	1.288
CZS 70	2.263	1.509	0.724
CZS 71	2.077	1.156	0.626
CZS 72	2.440	0.903	0.701
CZS 75	0.923	1.117	1.500
CZS 76	1.100	1.393	0.759
CZS 78	1.173	0.899	0.663
CZS 79	2.285	1.382	1.033
CZS 80	1.100	2.191	0.641
CZS 81	4.963	3.441	1.652
CZS 82	1.751	0.952	0.896
CZS 83	0.785	1.100	1.100
CZS 84	3.548	1.011	0.481
CZS 85	16.281	7.819	1.910
CZS 86	14.752	3.990	1.550
CZS 87	1.100	1.007	0.834
CZS 88	3.457	1.002	0.471
CZS 89	1.100	1.238	1.440
CZS 90	1.010	1.007	1.520
CZS 92	1.100	1.700	1.100
CZS 95	1.053	1.090	0.708
CZS 96	1.583	1.073	1.100
CZS 98	1.389	0.677	0.771
CZS 99	0.956	1.732	0.497
CZS 100	0.999	1.271	0.787
CZS 101	1.179	1.574	1.003
CZS 102	1.251	1.500	1.100
CZS 103	6.957	1.640	0.359
CZS 104	2.324	1.100	1.930
CZS 105	12.947	1.100	3.086
CZS 106	0.641	1.100	0.490
CZS 109	1.091	1.128	1.360
CZS 110	3.372	1.100	1.130
CZS 111	0.476	1.100	1.100
CZS 112	1.198	1.210	0.662
CZS 113	1.395	1.100	0.350
CZS 117	2.125	2.360	1.033
CZS 118	0.946	1.370	0.795
CZS 119	1.064	0.969	0.724
CZS 120	0.999	1.200	0.658
CZS 121	0.454	0.564	1.100
CZS 122	1.100	1.100	1.100
CZS 123	1.172	1.118	0.383
CZS 125	1.199	0.479	0.542
CZS 126	1.022	0.551	0.514
CZS 128	1.831	0.807	0.646

CZS 130	1.226	0.487	0.570
CZS 131	1.145	0.531	0.722
CZS 132	5.059	1.649	1.212
CZS 136	1.067	0.611	0.797
CZS 137	1.094	0.451	0.604
CZS 138	2.471	0.751	0.809
CZS 143	1.323	1.100	0.727
CZS 149	7.151	5.918	1.168
CZS 151	1.199	0.787	0.606
CZS 153	3.971	2.777	2.166
CZS 154	2.206	1.446	1.328
CZS 156	1.195	0.835	0.887
CZS 157	0.746	0.898	1.077
CZS 161	1.016	1.046	0.557
CZS 164	2.772	4.611	0.760
CZS 165	1.205	0.826	0.800
CZS 166	0.960	1.069	0.745
CZS 167	0.914	1.019	1.002
CZS 168	4.577	4.028	1.100
CZS 170	1.100	0.709	0.633
CZS 172	1.366	1.167	0.736
CZS 173	1.360	1.100	0.667
CZS 174	1.118	0.691	0.698
CZS 178	1.100	1.145	0.517
CZS 179	1.295	0.938	0.907
CZS 180	1.168	0.934	0.905
CZS 182	1.100	0.880	1.023
CZS 183	1.100	0.718	0.798
CZS 184	1.217	1.652	0.725
CZS 185	1.685	0.804	0.631
CZS 186	2.877	0.880	0.885
CZS 189	1.100	0.898	0.747
CZS 190	1.146	1.881	0.642
CZS 191	5.968	3.655	0.756
CZS 192	1.152	0.880	0.720
CZS 193	1.121	0.759	0.557
CZS 194	1.100	0.835	0.747
CZS 195	1.038	0.893	0.559
CZS 196	1.027	1.474	1.507
CZS 197	1.100	0.767	0.752
CZS 198	1.129	1.564	0.765
CZS 199	2.815	1.653	0.657
CZS 200	1.036	0.737	0.577
CZS 201	1.394	1.012	0.840
CZS 202	1.100	0.929	0.845
ML 1	1.132	0.665	0.601
ML 2	1.016	0.725	0.626
ML 3	1.049	0.936	0.806
ML 5	1.316	0.613	0.454
ML 6	1.100	1.100	1.006
ML 7	0.576	1.100	1.100
ML 8	6.524	0.765	1.045
ML 10	1.321	0.955	1.100
ML 13	2.751	1.123	1.100

ML 15	1.100	0.867	0.806
ML 17	0.931	1.069	1.100
ML 18	1.009	0.658	0.768
ML 22	1.100	0.801	1.100
ML 23	1.365	0.554	0.511
ML 24	1.100	1.065	0.768
ML 25	1.107	0.809	1.100
ML 26	2.075	1.333	1.076
ML 27	1.137	0.746	0.689
ML 28	0.511	1.195	0.459
ML 29	1.100	0.739	1.100
ML 31	4.652	0.833	1.100
ML 40	18.815	1.380	1.075
ML 41	3.930	1.326	1.147
ML 43	0.906	2.252	0.596
ML 44	12.500	5.249	1.670
ML 46	1.064	1.100	0.542
ML 47	1.256	1.100	0.822
ML 48	0.782	1.671	1.100
ML 49	0.462	1.100	0.652
ML 50	2.535	0.959	0.806
ML 51	13.648	2.699	1.382
ML 52	3.036	1.100	0.821
ML 53	1.186	1.100	0.634
ML 54	1.198	1.012	0.566
ML 55	1.880	0.902	0.887
ML 58	1.627	0.719	0.590
ML 60	1.350	0.567	0.566
ML 61	1.602	1.100	0.678
ML 64	1.795	0.821	0.596
ML 65	1.520	1.100	0.812
ML 66	1.093	0.886	0.790
ML 69	1.100	0.875	0.839
ML 70	5.218	1.100	0.739
ML 73	1.175	0.972	0.772
ML 74	4.070	1.085	1.259
ML 76	1.127	1.100	0.815
ML 77	13.912	3.815	1.066
ML 78	6.696	3.001	1.487
ML 79	1.100	0.630	0.775
ML 80	0.637	0.985	1.500
ML 82	1.003	0.580	0.721
ML 83	1.475	1.100	0.724
ML 84	1.100	1.100	0.710
ML 86	0.897	1.100	0.611
ML 87	1.120	1.100	1.239
ML 88	5.306	2.777	1.100
ML 89	1.201	0.755	0.770
ML 90	2.038	3.558	1.100
ML 91	4.230	1.282	0.616
ML 93	1.100	1.156	1.569
ML 94	1.256	0.994	0.796
ML 95	1.100	1.006	0.572
ML 96	1.231	0.713	0.536

ML 97	3.938	1.977	1.077
ML 98	1.069	0.719	0.696
ML 99	0.788	1.234	0.675
ML 100	1.157	1.462	1.076
ML 101	1.069	0.929	0.842
ML 103	1.263	0.653	0.872
ML 106	0.843	0.689	1.060
ML 107	1.161	0.702	1.034
ML 108	0.578	1.100	1.005
ML 109	0.942	1.114	0.528
ML 115	1.049	1.162	0.817
ML 119	1.100	0.905	0.569
ML 120	0.405	1.100	0.569
ML 121	0.303	1.100	0.397
ML 123	0.581	1.084	0.848
ML 124	1.829	1.492	1.168
GJ 1	10.220	3.330	1.698
GJ 3	6.920	1.100	0.912
GJ 4	1.183	0.645	0.688
GJ 5	1.176	1.100	0.710
GJ 6	1.016	0.592	0.761
GJ 7	1.576	0.835	0.725
GJ 8	5.844	1.130	0.725
GJ 9	1.415	1.100	0.710
GJ 10	9.775	1.100	0.860
GJ 11	1.564	0.811	1.003
GJ 12	1.475	0.611	0.772
GJ 14	1.324	0.857	1.100
GJ 15	1.009	0.948	0.801
GJ 16	1.100	0.807	0.518
GJ 19	1.100	0.770	0.767
GJ 21	1.968	1.661	0.959
GJ 22	1.212	0.683	0.737
GJ 23	2.028	1.907	0.908
GJ 24	1.020	0.652	0.710
GJ 25	1.243	0.633	1.140
GJ 26	1.188	1.237	0.707
GJ 28	1.128	1.012	1.002
GJ 29	1.628	1.100	0.719
GJ 30	1.316	0.683	0.856
GJ 31	1.100	0.626	0.730
GJ 33	1.244	0.773	0.833
GJ 34	1.003	0.616	0.851
GJ 35	1.440	0.504	0.645
GJ 36	1.096	0.502	0.749
GJ 37	1.100	0.766	0.900
GJ 38	2.008	1.180	0.774
GJ 39	1.132	0.724	0.710
GJ 40	0.852	0.759	1.030
GJ 41	0.648	1.100	1.100
GJ 42	1.120	1.003	0.829
GJ 43	0.990	1.100	0.928
GJ 44	1.591	1.100	1.100
GJ 45	1.100	0.586	0.793

GJ 46	1.001	0.588	0.957
GJ 48	1.100	1.100	0.738
GJ 49	1.100	0.542	1.005
GJ 50	1.832	1.471	1.183
GJ 51	1.100	1.292	1.540
GJ 52	1.100	1.038	0.738
GJ 53	1.100	0.647	0.599
GJ 56	1.060	0.599	2.010
GJ 57	1.496	0.891	0.781
GJ 58	1.672	0.872	0.722
GJ 59	1.876	0.641	0.769
GJ 60	1.100	1.435	1.106
GJ 61	2.550	0.891	1.100
GJ 62	7.966	1.100	0.777
GJ 63	1.488	0.579	0.797
GJ 64	2.251	1.100	2.090
GJ 65	1.596	0.744	0.743
GJ 67	3.793	1.036	0.846
GJ 70	1.197	0.663	1.220
GJ 73	4.026	0.651	0.870
GJ 75	0.855	0.747	1.002
GJ 80	1.574	0.773	1.026
GJ 81	1.039	1.100	0.724
GJ 82	1.050	0.749	0.641
GJ 83	1.007	0.912	0.757
GJ 84	3.194	0.656	0.815
GJ 85	2.160	1.608	1.508
GJ 86	1.004	0.731	0.886
GJ 87	29.853	9.374	3.393
GJ 88	1.100	1.054	0.742
GJ 90	1.212	1.966	1.141
GJ 91	0.777	0.783	1.141
GJ 94	1.115	0.608	0.624
GJ 96	1.127	0.546	0.872
GJ 97	0.958	0.799	1.241
GJ 99	1.100	1.100	1.212
GJ 100	1.106	0.855	0.918
GJ 102	1.027	0.526	0.882

Reactivity Index value was considered to classify individuals as positive (> 1) and negative (< 1).
